# Prophylactic Trimethoprim-Sulfamethoxazole for Allogeneic Hematopoietic Stem Cell Transplant Recipients During the Pre-engraftment Period

**DOI:** 10.1007/s44228-023-00029-7

**Published:** 2023-02-04

**Authors:** Kelly J. Gaffney, Theresa A. Urban, Mariana Lucena, Lisa Rybicki, Navneet S. Majhail, Sherif Beniameen Mossad

**Affiliations:** 1 Malignant Hematology & Blood Marrow Transplant, Medical University of South Carolina, 25 Courtenay Drive, Charleston, SC 29401 USA; 2 Cleveland Clinic, Cleveland, USA; 3 Medexus Pharmaceuticals, Bolton, Canada; 4 Sarah Cannon, Nashville, USA

**Keywords:** Hematopoietic transplant, Trimethoprim-sulfamethoxazole, Fluoroquinolone, Pre-engraftment

## Abstract

**Background:**

Our institution has used trimethoprim-sulfamethoxazole (TMP-SMX) as the antibacterial agent of choice for infection prophylaxis during the pre-engraftment period in the allogeneic transplant (allo-HCT) population.

**Methods:**

This retrospective, single center study was developed to compare the safety of that antibacterial prophylaxis to fluoroquinolones in allo-HCT. The primary endpoint was time to neutrophil engraftment.

**Results:**

A total of 366 patients were reviewed (TMP-SMX n = 332, fluoroquinolone *n* = 34)**.** No difference in days to neutrophil engraftment was found (median 15 versus 16 days, *p* = 0.62). Hyperkalemia was more common in the TMP-SMX cohort (32.2% versus 14.7%, *p* = 0.035); this did not contribute to a higher rate of agent discontinuation or arrhythmia. There was no significant difference in the incidence of neutropenic fever; however, those in the TMP-SMX cohort were more likely to have microbiologically confirmed bacteremia (24.1% versus 8.8% respectively, *p* = 0.043). There was no significant difference in infections. No long-term implication of prophylactic antibacterial agent selection was observed in terms of graft-versus-host-disease, underlying disease relapse, or mortality.

**Conclusion:**

The use of TMP-SMX was associated with a higher likelihood of bacteremia and hyperkalemia; however, this did not result in increased hospital stay, escalation of care, or mortality. The use of TMP-SMX for prophylaxis during the pre-engraftment period for allo-HCT recipients is safe and effective.

## Background

Patients who undergo a hematopoietic stem cell transplant (HCT) have a high probability of prolonged and profound neutropenia, increasing the risk of infection. Some guidelines recommend the use of antibacterial prophylaxis throughout the duration of neutropenia [1, 2]. Studies have outlined the benefits of antibacterial prophylaxis in the allogeneic HCT (allo-HCT) population, including improvements in infection-related and all-cause mortality [3]. Therefore, routine use of prophylaxis has become the standard of care at many institutions. Fluoroquinolone is the recommended first-line agent, as it is considered broad-spectrum with activity against *Pseudomonas aeruginosa* (PsA), and has been associated with lower incidence of gram-negative bacteremia [1, 4].

The prophylactic use of fluoroquinolones does not come without risks. They may select for microorganisms such as *Clostridium difficile* and enterococci [5, 6]. There is also concern for increasing rates of resistance against fluoroquinolones, which has been substantiated by various reports.

Finally, fluoroquinolones have unique toxicities, including retinal detachment and disabling damage to tendons, muscles, joints and the central nervous system [7].

For those who cannot tolerate a fluoroquinolone, an alternative agent such as trimethoprim-sulfamethoxazole (TMP-SMX) is recommended [2]. The use of TMP-SMX has been compared to fluoroquinolone use with no significant difference in infection-related or all-cause mortality identified [8]. TMP-SMX is not considered a first-line therapy option primarily due to its toxicity profile, myelosuppressive effects, and high rates of resistance development [8].

Our institution has preferentially used TMP-SMX over fluoroquinolones as the antibacterial agent of choice for infection prophylaxis during the pre-engraftment period in our allo-HCT population. The purpose of this report is to assess whether the continued use of TMP-SMX as our prophylactic agent of choice is both safe and effective.

## Methods

This retrospective, single center study was developed to compare the safety of TMP-SMX to fluoroquinolones when used as antibacterial prophylaxis in allo-HCT recipients. All infection-related data were captured in real-time by research personnel, and then reviewed and adjudicated by an infectious disease physician (SBM) before final entry into the institutional database. Additional data were collected by review of the electronic medical record.

All allo-HCT recipients prescribed either TMP-SMX or a fluoroquinolone for antibacterial prophylaxis were included. Patients received TMP-SMX 160–800 mg orally or intravenously every 12 h for antibacterial prophylaxis. This therapy was continued until time of engraftment or until alternative antibiotic therapy was clinically indicated in the setting of neutropenic fever or microbiologically targeted therapy. Fluoroquinolones were used only in those who could not tolerate TMP-SMX. Either levofloxacin 500 mg or ciprofloxacin 500 mg, dose-adjusted for renal function, per physician decision. Patient data were classified according to initial prophylactic agent chosen. Medical records were retrospectively reviewed from January 1, 2015 through December 31, 2019.

The primary endpoint was time to neutrophil engraftment. Safety endpoints included incidence of hyperkalemia, acute kidney injury, and QTc prolongation. Other endpoints included time to platelet engraftment, hospital length of stay, 30 day readmission rates, incidence of neutropenic fever, *Clostridium difficile* infection, other microbiologically-proven infection, escalation of clinical care through transfer to an intensive care unit, graft-versus-host-disease, underlying disease relapse, and mortality. Definitions of endpoints are described in Supplemental information.

Continuous variables were compared with Wilcoxon rank sum test. Categorical variables were compared with Chi-square or Fisher’s exact test. Outcomes were compared with log-rank test or Gray test.

## Results

**Fig. 1:**
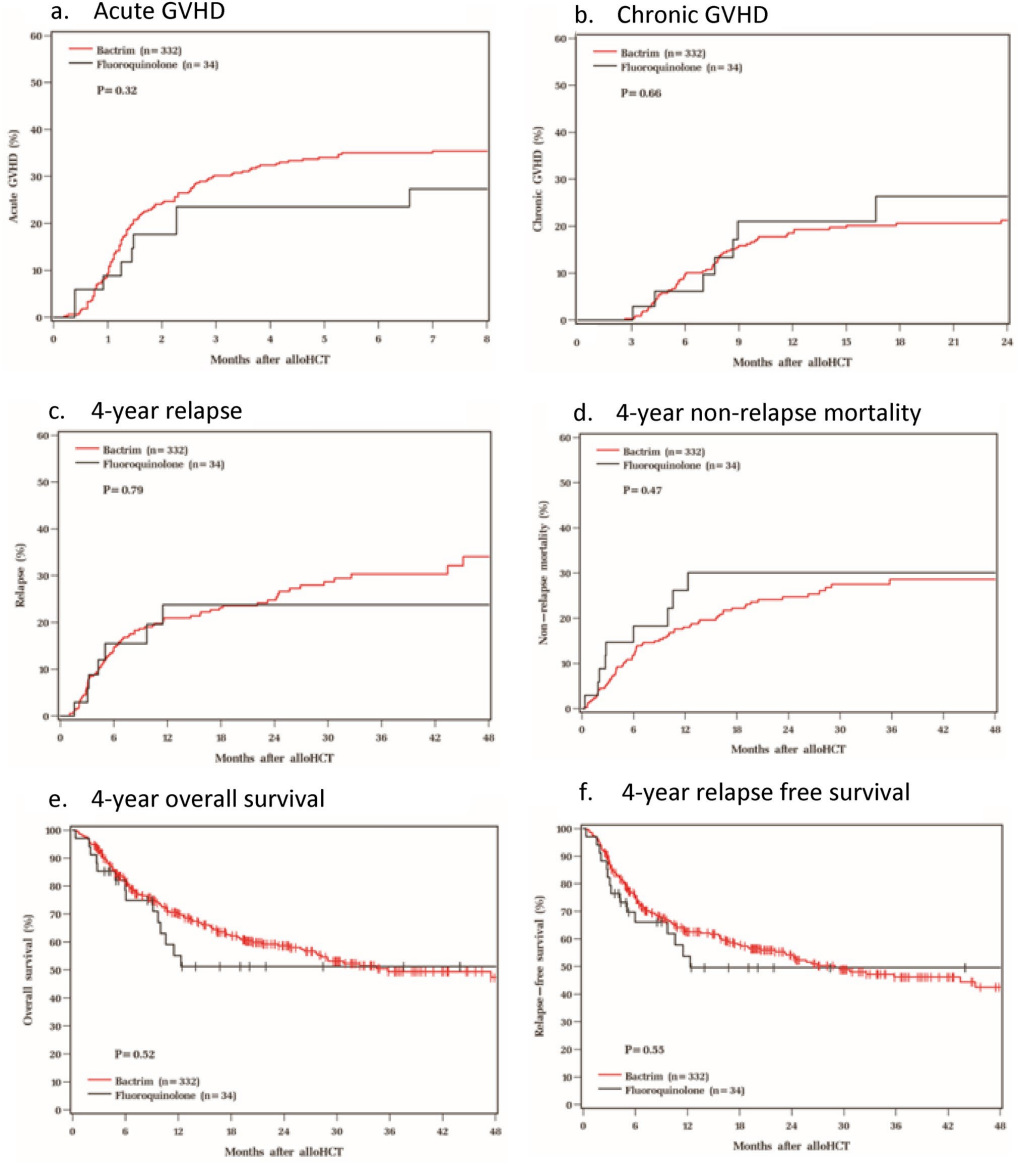
GVHD, relapse, mortality. **a** Acute GVHD. **b** Chronic GVHD. **c** 4-year relapse. **d** 4-year non-relapse mortality. **e** 4-year overall survival. **f** 4-year relapse free survival

Characteristics of the 366 patients are shown in Table 1. No difference in days to neutrophil engraftment between those who received TMP-SMX versus a fluoroquinolone was found (median 15 versus 16 days, *p* = 0.62). Hyperkalemia was more common in the TMP-SMX cohort (32.2% versus 14.7%, *p* = 0.035); this did not contribute to a higher rate of agent discontinuation or arrhythmia. There was no significant difference in the incidence of neutropenic fever; however, those in the TMP-SMX cohort were more likely to have microbiologically confirmed bacteremia (24.1% versus 8.8% respectively, *p* = 0.043). There was no significant difference in rates of PsA or other infections including urinary or respiratory tract infections. No long-term implication of prophylactic antibacterial agent selection was observed in terms of graft-versus-host-disease, underlying disease relapse or mortality. No significant differences were observed in any other secondary outcome as shown in Table 2 and Fig. 1.

**Table 1:** Baseline Characteristics

Variable	TMP-SMX (*n* = 332)		FQ (*n* = 34)		*p*-value
	*N*	%	*N*	%	
Age at transplant, years					0.75
Median (range)	60 (20–76)		60 (21–74)		
Gender					0.011
Male	183	55.1	11	32.4	
Female	149	44.9	23	67.6	
Karnofsky Performance Score					0.18
100	117	35.5	15	44.1	
90	149	45.2	9	26.5	
80	54	16.4	9	26.5	
70	10	3.0	1	2.9	
ECOG					0.85
0	133	40.2	16	47.1	
1	180	54.4	16	47.1	
2	17	5.1	2	5.9	
3	1	0.3	–	–	
Diagnosis					0.78
AML	167	50.3	18	52.9	
MDS	88	26.5	6	17.6	
ALL	19	5.7	3	8.8	
MFB	19	5.7	2	5.9	
CML	16	4.8	3	8.8	
Other	23	6.9	2	5.9	
Donor source					< 0.001
Matched unrelated donor	188	56.6	15	44.1	
UCB	11	3.3	7	20.6	
Haplo-identical	71	21.4	4	11.8	
HLA-identical sibling	62	18.7	8	23.5	
Graft source					< 0.001
PSC	183	55.1	16	47.1	
BM	138	41.6	11	32.4	
UCB	11	3.3	7	20.6	
Conditioning regimen					n/a
Myeloablative	99	29.8	11	32.4	
Reduced intensity	233	70.2	23	67.6	
GVHD prophylaxis					n/a
FK/MMF	95	28.6	7	20.6	
FK/MMF/PTCY	94	28.3	8	23.5	
CSA/MMF	47	14.2	9	26.5	
FK/MTX	50	15.1	6	17.6	
FK/MMF/MTX	27	8.1	2	5.9	
FK/MTX/Bortezomib	7	2.1	1	2.9	
FK/MTX/Maraviroc	5	1.5	–	–	
FK	4	1.2	–	–	
FK/ATG	1	0.3	1	2.9	
CSA/MMF/MTX	1	0.3	–	–	
Sirolimus/MMF/PTCY	1	0.3	–	–	

*TMP-SMX* trimethoprim-sulfamethoxazole, *FQ* fluoroquinolone, *ECOG* Eastern Cooperative Oncology Group, *AML* acute myeloid leukemia, *MDS* myelodysplastic syndrome, *ALL* acute lymphoblastic leukemia, *MFB* myelofibrosis, *CML* chronic myeloid leukemia, *NHL* Non-Hodgkin’s lymphoma, *MPN* myeloproliferative neoplasm, *PLL* prolymphocytic leukemia, *PNH* paroxysmal nocturnal hemoglobinuria, *CLL* chronic lymphoblastic leukemia, *HL* Hodgkin’s lymphoma, *UCB* umbilical cord blood, *PSC* peripheral stem cell, *BM* bone marrow, *FK* tacrolimus, *MMF* mycophenolate mofetil, PTCY: post-transplant cyclophosphamide, *CSA* cyclosporine, *MTX* methotrexate

**Table 2:** Outcomes

Variable	TMP-SMX (*n* = 332)		FQ (*n* = 34)		*p*-value
	*N*	%	*N*	%	
Time to neutrophil engraftment, days					0.62
Median (range)	15 (10–44)		16 (6–40)		
Time to platelet engraftment, days					0.97
Median (range)	26 (10–175)		25 (11–166)		
Length of stay from admission, days					0.77
Median (range)	27 (17–95)		27 (18–124)		
Length of stay from transplant, days					0.92
Median (range)	20 (8–90)		20 (10–117)		
Clinical events					
Neutropenic fever	160	48.2	18	52.9	0.60
Escalation of antibiotics	204	61.4	20	58.8	0.77
Bacteremia	80	24.1	3	8.8	0.043
Pseudomonas bacteremia	3/79	3.8	0/3	0	1.0
UTI	14	4.2	4	11.8	0.07
RTI	6	1.8	2	5.9	0.16
Other infection	6/82	7.3	1/5	20.0	0.35
C. Diff infection	41	12.3	1	2.9	0.15
Hospital readmission	29	8.7	2	5.9	0.75
Escalation to ICU	40	12.0	8	23.5	0.10
Safety endpoints					
Hyperkalemia	107	32.2	5	14.7	0.04
Acute kidney injury	172	51.8	18	52.9	0.90
Prolonged QTc	32/202	15.8	6/20	30.0	0.12
Intolerance to antimicrobial agent	4	1.2	0	0	1.0
Acute GVHD, worst grade					–
0	214	64.5	25	73.5	
1	36	10.8	3	8.8	
2	53	16.0	3	8.8	
3	18	5.4	2	5.9	
4	11	3.3	1	2.9	
Chronic GVHD, worst extent					–
None	270	81.3	27	79.4	
Limited	13	3.9	4	11.8	
Extensive	49	14.8	3	8.8	
Disease relapse	82	24.7	7	20.6	–
Status at last follow-up					
Alive	201	60.5	20	58.8	
Dead	131	39.5	14	41.2	–
Non-relapse mortality	76	58.0	9	64.3	

*TMP-SMX* trimethoprim-sulfamethoxazole, *FQ* fluoroquinolone, *SD* standard deviation, *UTI* urinary tract infection, *RTI* respiratory tract infection, *GVHD* graft-versus-host-disease, *ICU* intensive care unit

## Discussion and Conclusion

Our study showed prophylactic TMP-SMX does not delay engraftment in allo-HCT recipients. We acknowledge that our small sample size may not have detected such differences given the rare incidence of TMP-SMX induced myelosuppression. The cohort of patients who received fluoroquinolone prophylaxis were more likely to have an UCB graft source; this is likely due to a clinical trial actively enrolling patients using UCB during the time period patients’ data were collected. That trial prohibited any use of TMP-SMX. Although time to engraftment is typically longer with an UCB source, the UCB graft source used in that clinical trial showed significantly shorter time to engraftment compared to engraftment following transplants using traditional UCB grafts [9]. A majority of patients received reduced-intensity conditioning regimens and may not have as profound or extended cytopenias as those who receive myeloablative chemotherapy.

Drug-specific side effects observed in our patients were aligned with what one would expect with each agent and did not contribute to a difference in either agent discontinuation rates. The low number of reviewed patients who received fluoroquinolones may have increased the risk of not capturing all adverse effects of the agent.

One major concern with TMP-SMX is its lack of activity against PsA. The use of prophylactic agents with anti-pseudomonal activity has reduced the number of documented gram-negative infections and shown survival benefit [3, 10]. We observed no difference in the rate of PsA infection between those who received TMP-SMX and those who received ciprofloxacin. This may be due to the low incidence of PSA infection observed in our patients compared to historical reports [11].

Antibiotics have been associated in varying degrees with the development of *Clostridium difficile* infection (CDI). The risk of CDI with fluoroquinolone use is thought to be greater than that seen with TMP-SMX [12]. The higher rates of CDI observed in this TMP-SMX cohort may be due to limited sample size. Additionally, CDI was defined as a positive polymerase chain reaction (PCR) test. In the summer of 2018, a new two-step process for *Clostridium difficile* testing was implemented at this center and all positive PCR specimens had confirmatory toxin enzyme immunoassay (EIA) tests performed. However, as not all included patients were hospitalized after this two-step process was implemented, the EIA data were not compared between the two groups. The number of CDI reported in either cohort may be an overestimation, due to the moderate positive predictive value of the PCR test.

The higher number of infections resistant to TMP-SMX is consistent with reports from other centers. It is not well understood why this is observed but may have to do with the difference in spectrum of activity of these agents. The rate of breakthrough bloodstream infections with TMP-SMX should be further explored to ensure the use of this agent is beneficial.

This retrospective analysis did not detect a difference in the time to neutrophil and platelet engraftment in allo-HCT recipients who received TMP-SMX or a fluoroquinolone for antibacterial prophylaxis. Although the use of TMP-SMX was associated with a higher likelihood of bacteremia and hyperkalemia, this did not result in increased hospital length of stay, escalation of care, or mortality. Importantly, TMP-SMX use did not impact the time to or rate of engraftment. The use of TMP-SMX for antimicrobial prophylaxis during the pre-engraftment period for allo-HCT recipients is safe and effective.

## Data Availability

The data that support the findings of this study are available from the corresponding author, KG, upon reasonable request.
